# A novel mitochondrial DNA variant in *MT-ND6:* m.14430A>C p.(Trp82Gly) identified in a patient with Leigh syndrome and complex I deficiency

**DOI:** 10.1016/j.ymgmr.2024.101078

**Published:** 2024-03-29

**Authors:** Surita Meldau, Sally Ackermann, Gillian Riordan, George F. van der Watt, Careni Spencer, Sharika Raga, Kashief Khan, Dee M. Blackhurst, Francois H. van der Westhuizen

**Affiliations:** a National Health Laboratory Service, Groote Schuur Hospital, Cape Town, South Africa; b Division of Chemical Pathology, University of Cape Town, Cape Town, South Africa; c Private Practice, Constantiaberg Mediclinic, Cape Town, South Africa; d Division of Paediatric Neurology, Dept of Paediatrics and Child Health, University of Cape Town, Red Cross War Memorial Children's Hospital, Cape Town, South Africa; e Department of Medicine, Groote Schuur Hospital and Division of Human Genetics, University of Cape Town, Cape Town, South Africa; f Neuroscience Institute, University of Cape Town, South Africa; g International Centre for Genomic Medicine in Neuromuscular Diseases Study, University College London, London, United Kingdom; h Focus Area for Human Metabolomics, North-West University, Potchefstroom, South Africa

**Keywords:** *MT-DN6*, Leigh syndrome, Novel mtDNA variant, Complex I deficiency

## Abstract

Leigh syndrome is a severe progressive mitochondrial disorder mainly affecting children under the age of 5 years. It is caused by pathogenic variants in any one of more than 75 known genes in the nuclear or mitochondrial genomes.

A 19-week-old male infant presented with lactic acidosis and encephalopathy following a 2-week history of irritability, neuroregression and poor weight gain. He was hypotonic with pathological reflexes, impaired vision, and nystagmus. Brain MRI showed extensive bilateral symmetrical T2 hyperintense lesions in basal ganglia, thalami, and brainstem. Metabolic workup showed elevated serum alanine, and heavy lactic aciduria with increased ketones, fumarate, malate, and alpha-ketoglutarate as well as reduced succinate on urine organic acid analysis. Lactic acidemia persisted, with only a marginally elevated lactate:pyruvate ratio (16.46, ref. 0–10). He demised at age 7 months due to respiratory failure.

Exome sequencing followed by virtual gene panel analysis for pyruvate metabolism and mitochondrial defects could not identify any nuclear cause for Leigh syndrome. Mitochondrial DNA (mtDNA) genome sequencing revealed 88% heteroplasmy for a novel variant, NC_012920.1(MT-ND6):m.14430A>C p.(Trp82Gly), in blood DNA. This variant was absent from the unaffected mother's blood, fibroblast, and urine DNA, and detected at a level of 5% in her muscle DNA.

Mitochondrial respiratory chain analysis revealed markedly reduced mitochondrial complex I activity in patient fibroblasts (34% of parent and control cells), and reduced NADH-linked respirometry (less than half of parental and control cells), while complex II driven respirometry remained intact. The combined clinical, genetic, and biochemical findings suggest that the novel MT-ND6 variant is the likely cause of Leigh syndrome in this patient. The mitochondrial ND6 protein is a subunit of complex I.

An interesting finding was the absence of a significantly elevated lactate:pyruvate ratio in the presence of severe lactatemia, which directed initial diagnostic efforts towards excluding a pyruvate metabolism defect. This case highlights the value of a multidisciplinary approach and complete genetic workup to diagnosing mitochondrial disorders in South African patients.

## 1 Introduction

Leigh syndrome (subacute necrotizing encephalopathy) is a severe progressive mitochondrial disorder occurring most commonly in infants and young children. Rarely cases may present in adulthood. It is characterised by progressive psychomotor degeneration with characteristic symmetrical T2 hyperintensities in the basal ganglia, thalamus, substantia nigra and/or brainstem. The age of onset is usually under 5 years, with most patients presenting between the ages of 3 and 12 months [[1], [2], [3]].

More than 100 different genes have been associated with Leigh syndrome in the literature, more than half of which have either definitive or moderate disease associations according to a recent Clinical Genomics (Clingen) expert panel review [1]. Most of these genes either encode essential proteins that make up the human oxidative phosphorylation (OXPHOS) system, or proteins responsible for the assembly, stability and functioning of these components. Fifteen of these genes are located on the mitochondrial DNA (mtDNA) genome. Known pathogenic variants in these mtDNA genes account for only about 10–20% of Leigh cases worldwide, while the remainder are caused by mutations in a growing number of nuclear genes [1,4].

Here we describe a novel, likely pathogenic variant in *MT-ND6*, encoding the ND6 subunit of complex I in a patient with Leigh syndrome.

## 2 Case report

The patient, a 19-week-old male infant, presented to a local paediatrician with compensated lactic acidosis and encephalopathy. His parents reported persistent irritability following his 14-week vaccines, as well as a 2-week history of lethargy, poor weight gain and neuroregression, including loss of head control, eye contact and social smile.

He was the first child born to non-consanguineous, healthy parents. Routine paediatric visits at 6 and 14 weeks found normal development, except for mild head lag.

Upon admission, he was hypotonic with significant head lag, pathological tendon reflexes and intermittent dystonic posturing. He displayed no social interactions or visual response to light. He had sluggish pupillary responses, an intermittent divergent squint and nystagmus. Fundoscopy was normal. His respiratory drive was impaired with a tendency to over-compensate in the presence of physiological stress or infection.

**Fig. 1:**
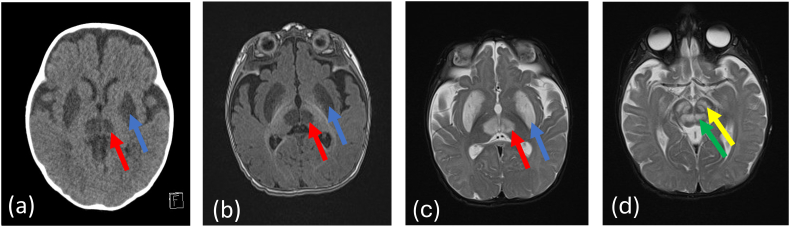
(a) Axial CT and (b) MRI T1 weighted imaging showing hypointensity of the putamina (blue arrows) and thalami (red arrows); (c) T2 hyperintensity of the putamina (blue arrows) and thalami (red arrows); (d) T2 showing midbrain lesions in the substantia nigra (yellow arrow) and red nuclei (green arrow).

An initial uncontrasted CT scan showed bilateral hypodensities in the basal ganglia and brainstem. Brain MRI showed extensive bilateral symmetrical T2 hyperintense and T1 hypointense lesions in the basal ganglia, thalami, and brainstem. See Fig. 1.

**Fig. 2:**
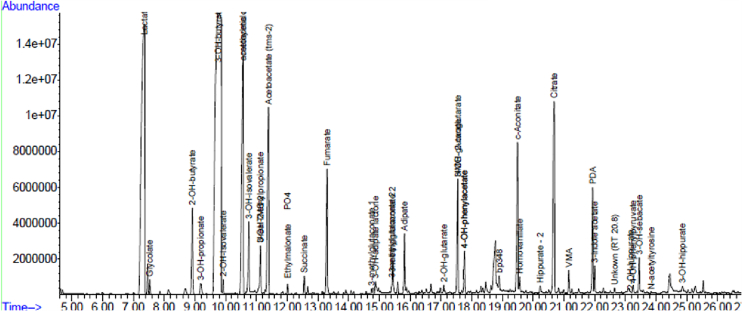
Urine organic acid findings indicating significant lactaturia and elevated ketones (3OH butyric acid and acetoacetate), relative to the internal standard (PDA), and elevated Krebs cycle intermediates with a reverse succinate/fumarate ratio.

Initial blood gas showed lactatemia (serum lactate of 8.9 mmol/L, ref. 0.5–2.2 mmol/L), while urine organic acid analysis by GC–MS revealed heavy lactaturia and ketonuria, together with elevated fumarate, malate and alpha-ketoglutarate, and a reversed succinate to fumarate ratio (see Fig. 2). In addition, elevated serum alanine (679 μmol/L, ref. 144–348 μmol/L) was found on serum amino acid analysis.

The serum lactate to pyruvate ratio was only marginally elevated (16.46, ref. 0–10), which in the presence of the high lactate, as well as the clinical picture and neuroimaging, suggested a possible defect of pyruvate metabolism as the cause of the Leigh syndrome in this patient.

A high fat, restricted carbohydrate diet was introduced (ketogenic diet), providing ketone bodies as an alternative fuel to the tricarboxylic acid cycle via beta-oxidation. The patient's lactate levels initially responded well but he developed refractory keto-acidosis once serum ketones rose above 2 mmol/L.

A percutaneous endoscopic gastrostomy was inserted for enteral feeding, and he severely decompensated post-operatively requiring non-invasive respiratory support. Further metabolic decompensation occurred after urgent surgery for an incarcerated inguinal hernia a few weeks later. He demised at the age of 7 months due to respiratory failure.

## 3 Methods

### 3.1 Fibroblast culture

Skin biopsies were obtained from the affected patient and both parents, and fibroblast cultures were established.

### 3.2 Genetic investigations

#### 3.2.1 mtDNA deletion screening and sequencing

Comprehensive mtDNA analysis for the proband was done through the National Health Laboratory Services Inherited Metabolic Diseases diagnostic laboratory in Cape Town, South Africa. Briefly, mtDNA deletion analysis consisted of long-range PCR covering the entire coding region of the mtDNA genome, followed by agarose gel electrophoresis to detect single or multiple mtDNA deletions or rearrangements. This amplicon was used for library preparation using the Nexterra XT DNA library prep kit (Illumina, UK), followed by next generation sequencing using the MiSeq Reagent Kit v2 Micro (300 cycle) (Illumina, UK) on the Illumina MiSeq platform. Data analysis was performed using the mtDNA server [5]. This process was followed using both the proband's leukocyte DNA, as well as leukocyte, skin fibroblast, and muscle DNA from the proband's mother, and muscle DNA from the maternal grandmother.

The presence of the detected variant of interest was further investigated using Sanger sequencing in both leukocyte, urine, and skin fibroblast DNA from the proband, and leukocyte and fibroblast DNA from the proband's mother.

#### 3.2.2 nDNA investigations

Exome sequencing was performed on DNA from the proband using the Ion Ampliseq Exome RDY kit (Thermo Fisher Scientific, USA) to rule out nDNA- inherited Leigh syndrome or more specifically PDH deficiency, given the low serum lactate to pyruvate ratio despite severe lactatemia. Virtual panels for analysis were compiled using the Genomics England PanelApp Pyruvate Dehydrogenase (v.1.2) and Mitochondrial Disorders (v.2.107) panels [6].

### 3.3 Mitochondrial respiratory chain enzymology

Respiratory chain enzyme activities were measured in enriched mitochondrial preparations from fibroblasts. Briefly, fibroblasts from 4 × 75 cm^2^ flasks were suspended in 10 mM Tris.HCl, pH 7.6 and following homogenization with a tight-fitting Dounce homogenizer, a mitochondrial preparation was prepared by differential centrifugation at 4 °C as described by Janssen et al. [7]. Using the suspended mitochondria pellets, CI (measured as NADH oxidation), CII, CIII and CIV were measured individually based on kinetic methods as described before [2]. Activities were normalized to total protein content, and additionally CI relative to CII, CIII and CIV.

### 3.4 Fluorespirometry

To test the maximum mitochondrial respiratory capacity in these fibroblasts, the O2k-FluoRespirometer (Oroboros Instruments, Innsbruck, Austria) was used, following the SUIT 008 O2 ce-pce D025 protocol [8]. Freshly prepared fibroblasts were suspended in MIRO5 buffer at 1 × 10^6^ cells per mL and the substrates and inhibitors addition injected according to the protocol. This enabled an assessment of the N- and S-pathways in the Q-junction, and thus a physiologically estimate of the maximum mitochondrial respiratory capacity.

## 4 Results

### 4.1 Genetic investigations

#### 4.1.1 mtDNA sequencing

**Fig. 3:**
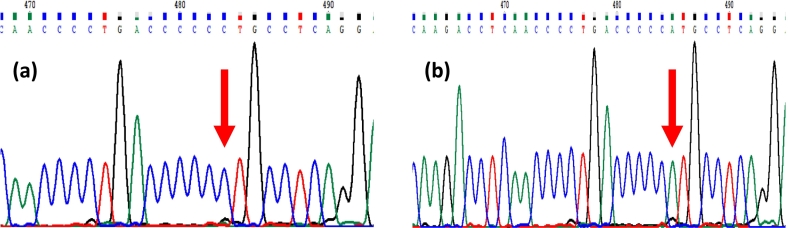
DNA sequence electropherograms of part of the *MT-ND6* gene in (a) the patient blood DNA and (b) maternal skin fibroblast DNA. The site of the m.14430 A > C variant is indicated with an arrow.

NGS sequence of the mtDNA coding region was obtained with an average coverage depth of 7962×. A single variant of interest was identified in the mtDNA from the proband's peripheral blood. The MT-ND6:m.14430A>C p.(Trp82Gly) was present at 88.2% heteroplasmy (5780 reads) in blood leukocytes (mitochondrial haplogroup H48). Although not quantitated, similarly high levels were estimated in the patient's skin fibroblast and urine DNA on Sanger sequencing (see Fig. 3).

The m.14430A>C variant was undetectable in DNA from the mother's peripheral blood (0% of 1501 reads), and skin fibroblasts (0% of 1704 reads), however, upon further investigation to refine reproductive risks, was detected at low heteroplasmy (5% of 3394 reads) in her muscle DNA. On clinical examination and history, the mother had no features of concern. Subsequent analysis of the maternal grandmother's muscle DNA was unable to detect any copies of this variant (0% of 3576 reads), suggesting a de novo occurrence in the mother. Both mother and grandmother were shown to have the same haplogroup, H48, as the affected child.

#### 4.1.2 nDNA investigations

No candidate variants were identified in exome data from this patient in any of the nDNA genes represented in the virtual PDH deficiency or Mitochondrial Disorders panels investigated, making a nDNA cause of disease less likely.

### 4.2 Mitochondrial respiratory chain enzymology and respirometry

The respective respiratory chain enzymes (CI-IV) in the proband were measured in enriched mitochondria preparations (Table 1). Compared to the mean of activities of the parent and control cells, which were comparable, the CI activity of the proband were markedly lower when normalized to protein content (34%), as well as the ratios with CII (31%), CIII (28%) and CIV (42%). This observation was reflected in the NADH-linked (CI) respirometry in digitonin-permeabilized fibroblasts. This is particularly noticeable with glutamate-stimulated respirometry, where the oxygen consumption rate was less than half the rate of the parent and control fibroblasts. Of further interest was the much more comparable succinate-stimulated oxygen consumption rate between the proband and parent and control cells, confirming the enzyme data that CII-driven respirometry is not affected in the proband.

**Table 1:** Respiratory chain enzyme activities and high resolution respirometry in fibroblasts.

	CI	CII	CIII	CIV	CI/II	CI/CIII	CI/CIV	PM	G	S
	μmol/min/μg							pmol/s/mL		
Patient	4.0 ± 0.4	28.1 ± 0.8	34.9 ± 1.2	21.9 ± 0.8	0.14	0.11	0.18	3.95	27.5	83.6
Mother	12.2 ± 1.6	26.9 ± 0.4	36.9 ± 1.7	30.2 ± 0.9	0.45	0.33	0.40	5.25	73.4	98.0
Father	12.5 ± 2.5	27.1 ± 0.4	26.7 ± 0.6	24.9 ± 0.7	0.46	0.47	0.50	4.72	54.2	68.3
Control	10.4 ± 1.1	23.6 ± 0.7	25.5 ± 0.8	29.2 ± 4.4	0.44	0.41	0.36	5.65	77.3	89.8

Enzyme activities are normalized to total mitochondrial protein content, or individual ratios, as mean (*n* = 3) ± SD. PM (pyruvate, malate), G (glutamate) and S (succinate) indicate relevant high resolution respirometry substrates in permeabilized fibroblasts (10^6^ per mL reaction).

## 5 Conclusions

Leigh syndrome is the most common cause of mitochondrial disease in children and is frequently associated with mitochondrial complex I deficiency, with genetic defects reported in 22 complex I subunits, and 8 complex I assembly factors known associated with this disease.

The novel m.14430A>C variant identified in this patient’s mtDNA results in a Tryptophan to Glycine change at position 82 of the NADH dehydrogenase (complex I) subunit 6 protein. At least six other variants in *MT-ND6* have been associated with Leigh syndrome, including the m.14430A>G, p.(Trp82Arg) variant recently reported in a patient with complex 1 deficiency, affecting the same nucleotide with a different amino acid change [9].

This variant is absent from population databases, including Genbank, GnomAD, and Helix, is predicted to be likely pathogenic by Apogee2 (pathogenicity score of 0.854, probability: 0.977) [10], and occurs in a very conserved region of the mtDNA genome (97.8%). No causative variants were identified in exome data for any of the Leigh syndrome genes included in the Genomics England PanelApp genes listed for this disorder.

We have furthermore shown that complex 1 enzyme activity and complex 1 driven respiration was reduced in the patient's fibroblasts compared with unaffected parental and control cell lines.

The high heteroplasmy levels seen in the affected patient's blood, urine, and fibroblast samples, in contrast to the low level (5%) in the unaffected mother's muscle DNA, and the complete absence of the variant in the unaffected maternal grandmother's muscle tissue, and the mother's urine and fibroblast cells, further supports pathogenicity. This variant likely arose de novo in the proband's mother.

The findings presented in the manuscript, strongly support pathogenicity of the m.14430 A > C variant in *MT-ND6* as a cause of Leigh syndrome in this patient.

## Funding

This work was supported by the National Health Laboratory Service Research Trust [grant number 00494667].

## CRediT authorship contribution statement

**Surita Meldau:** Writing – review & editing, Writing – original draft, Project administration, Methodology, Investigation, Funding acquisition, Formal analysis, Data curation, Conceptualization. **Sally Ackermann:** Writing – review & editing, Investigation. **Gillian Riordan:** Writing – review & editing, Supervision, Investigation. **George F. van der Watt:** Investigation, Formal analysis, Data curation. **Careni Spencer:** Investigation. **Sharika Raga:** Investigation. **Kashief Khan:** Investigation. **Dee M. Blackhurst:** Supervision. **Francois H. van der Westhuizen:** Writing – review & editing, Methodology, Investigation, Formal analysis, Data curation, Conceptualization.

## Declaration of competing interest

The authors have no conflicts of interest.

## Data Availability

Data may be will be made available on request, where specific consent from the family allows.
